# A metacognitive approach to the study of motion-induced duration biases reveals inter-individual differences in forming confidence judgments

**DOI:** 10.1167/jov.23.3.15

**Published:** 2023-03-27

**Authors:** Aurelio Bruno, Jennifer Sudkamp, David Souto

**Affiliations:** 1 Department of Psychology, University of York, York, UK; 2 School of Psychology and Vision Sciences, College of Life Sciences, University of Leicester, Leicester, UK; 3 School of Psychology and Vision Sciences, College of Life Sciences, University of Leicester, Leicester, UK; 4 School of Psychology and Vision Sciences, College of Life Sciences, University of Leicester, Leicester, UK

**Keywords:** metacognition, time perception, speed, individual differences, computational modeling

## Abstract

Our ability to estimate the duration of subsecond visual events is prone to distortions, which depend on both sensory and decisional factors. To disambiguate between these two influences, we can look at the alignment between discrimination estimates of duration at the point of subjective equality and confidence estimates when the confidence about decisions is minimal, because observers should be maximally uncertain when two stimuli are perceptually the same. Here, we used this approach to investigate the relationship between the speed of a visual stimulus and its perceived duration. Participants were required to compare two intervals, report which had the longer duration, and then rate their confidence in that judgment. One of the intervals contained a stimulus drifting at a constant speed, whereas the stimulus embedded in the other interval could be stationary, linearly accelerating or decelerating, or drifting at the same speed. Discrimination estimates revealed duration compression for the stationary stimuli and, to a lesser degree, for the accelerating and decelerating stimuli. Confidence showed a similar pattern, but, overall, the confidence estimates were shifted more toward higher durations, pointing to a small contribution of decisional processes. A simple observer model, which assumes that both judgments are based on the same sensory information, captured well inter-individual differences in the criterion used to form a confidence judgment.

## Introduction

Deciding about the relative duration of two or more brief visual events can be influenced by both purely visual manipulations, such as visual adaptation (Bruno & Cicchini, 2016; Johnston, Arnold, & Nishida, 2006), and more cognitive factors, such as attention (Cicchini & Morrone, 2009; Pariyadath & Eagleman, 2007; Tse, Intriligator, Rivest, & Cavanagh, 2004). To some extent, the formulation of any visual duration judgment requires contributions from both sensory and cognitive components, but the relevance of each component in forming that judgment is often difficult to estimate. More specifically, we still do not know how to assess the perceptual nature of duration distortions, disentangling it from the cognitive interpretation of the elicited sensations, which can also affect our decisions.

In this study, we were interested in the effect of visual motion on duration judgments. The ability to accurately estimate the duration of an object that moves at a constant or changing speed in our visual environment is essential in many everyday activities. For example, when we need to cross the road, to avoid a collision we need to be able to estimate the time to arrival of approaching cars or bicycles, integrating, among other things, information regarding their driving speed. As much as we can perform the task in this context (Sudkamp, Bocian, & Souto, 2021), the temporal metrics for perceived duration and anticipatory actions were shown to be dissociable (Marinovic & Arnold, 2012). In fact, when we are required to compare objects moving at different speeds, our perception of their duration is often biased. A moving visual object is perceived to last longer than a stationary object of equal duration and with otherwise identical features (Brown, 1931; Goldstone & Lhamon, 1974; Kanai, Paffen, Hogendoorn, & Verstraten, 2006; Kaneko & Murakami, 2009; Lhamon & Goldstone, 1974; Roelofs & Zeeman, 1951). Also, the duration of an interval containing a visual stimulus with increasing or decreasing speed over time is judged to be different from that of an interval that embeds an identical stimulus moving at a constant speed (Binetti, Lecce, & Doricchi, 2012; Binetti, Tomassini, Friston, & Bestmann, 2020; Bruno, Ayhan, & Johnston, 2015; Matthews, 2011; Sasaki, Yamamoto, & Miura, 2013), even when the two stimuli have the same average speed. At the same time, the precision of duration judgments remains constant across different speed profiles, indicating that differences in speed affect perceived duration but not duration discrimination. These results support the idea, proposed by a content-sensitive clock model (Johnston, 2010; Johnson, 2014) and by a neural network model (Roseboom et al., 2019), that the sensory content of an interval (for example, the speed profile of a stimulus) and not just its onset and offset is taken into account when a decision about its duration is formulated in our brain. Beyond biases in sensation, however, there might as well be a contribution of more decisional factors, such as if participants base their response on an irrelevant feature of the stimulus. These could introduce biases in the response even if perception was in fact unaffected.

Our ability to reflect on our own performance allows us to assign levels of confidence to our decisions. These self-evaluations contribute to guide our future behavior (Lisi, Mongillo, Milne, Dekker, & Gorea, 2021). Traditionally, a confidence judgment has been thought to reflect our beliefs in the correctness of these decisions. In the visual modality, much attention has been focused on how confidence evaluations are informed by perception (Arnold, Saurels, Anderson, & Johnston, 2021; de Gardelle & Mamassian, 2014; Mamassian & de Gardelle, 2021; Song, Kanai, Fleming, Weil, Schwarzkopf, & Rees, 2011; Spence, Dux, & Arnold, 2016; Zylberberg, Barttfeld, & Sigman, 2012). An alternative view is that confidence reflects instead the consistency of our perceptual experience (Caziot & Mamassian, 2021).

It has been recently proposed that the way confidence judgments map onto performance in perceptual tasks might disambiguate between perceptual biases and systematic decision biases (Gallagher, Suddendorf, & Arnold, 2019). The authors adapted two groups of participants to either coherent motion or random motion and instructed only the latter group to always provide the same response when uncertain about the motion direction of the test stimulus. In this way, the shift in the reported direction of motion in the condition with random motion (which was purely “decisional”) was found to be comparable to that induced by adapting to coherent motion, but the uncertainty peaks (i.e., stimulus levels that elicited minimal confidence) were aligned with performance only after adaptation to coherent motion, revealing a dissociation between the two measures. This means that, when the observed effect is little or not affected by decisional factors, confidence judgments can be equally good estimators of perceptual changes as discrimination judgments. The assumption is that, when judgments are primarily based on sensory information, our confidence in those judgments should be minimal when there is no sensory difference being detected (i.e., when we see the two stimuli as being identical along the dimension of interest). On the other hand, when the judgments are subject to a decisional bias, we would expect a dissociation between the point of minimal confidence and the point where the two stimuli are seen to be identical. For example, when required to compare the duration of two stimuli that appear equally long, we may repeatedly respond that the faster stimulus is of a shorter duration, inducing a bias in discrimination, but our confidence in those judgments would still be minimal at this point, indicating that the distortion is primarily due to a decisional but not a sensory difference.

So far, this approach has been employed in various contexts, such as to show that implied motion aftereffect depends more on decision making than perceptual processes (Gallagher, Suddendorf, & Arnold, 2021), to rule out a gaze-contingent response bias in the effect of pursuit eye movements on perceived background motion (Luna, Serrano-Pedraza, Gegenfurtner, Schutz, & Souto, 2021), or to demonstrate that both motor and sensory adaptation influence numerosity perception at a pre-decisional stage (Maldonado Moscoso, Cicchini, Arrighi, & Burr, 2020). Also, confidence estimates were shown to closely follow perceptual decisions both after sensory adaptation (to orientation or color) and after manipulation of prior statistics of the presented stimuli (Caziot & Mamassian, 2021). To our knowledge, however, no study has yet approached duration perception using the same method.

Here, in two experiments, we asked our participants to compare the relative duration of two sequentially displayed visual intervals, one of them containing a stimulus with the same duration (500 ms) across trials and different speed profile across conditions (drifting at a constant rate, stationary, accelerating or decelerating) and the other containing a stimulus with variable duration and drifting at a constant speed. Participants were required to judge the relative duration of the two intervals and then rate their confidence in the correctness of their decision. We estimated, for each speed profile, the point of perceived equality of the durations of the two stimuli and the point that elicited the minimum confidence, and we used them as our measure of perceived duration as estimated by discrimination and confidence, respectively. We then compared the alignment between these two measures. We found that both measures were affected by stimulus speed in the expected direction (i.e., strong duration compression for stationary stimuli, milder compression for accelerating and decelerating stimuli). However, the magnitude of these shifts was overall smaller for confidence, indicating a small contribution of decisional processes to the effect of speed on perceived duration. Finally, we describe the predictions of a simple observer model, which assumes that both types of judgments depend on the discriminability of the same sensory signals but that high confidence requires an internal criterion (Arnold et al., 2021; Mamassian & de Gardelle, 2021) to be exceeded. We found that we can use this criterion to account for inter-individual differences in confidence and to estimate the contributions of different components to the formation of a confidence judgment.

## General methods

### Participants

Two separate groups of observers took part in either Experiment 1 or Experiment 2. Participants were either University of York students and participated for course credit or were recruited through Prolific (Palan & Schitter, 2018) at https://www.prolific.co/ and received monetary compensation for their participation (£7 per hour). We selected participants who were fluent in the English language and who reported normal or corrected-to-normal vision. For Experiment 2, we also restricted the age range for participation to 18 to 50 years old. The study was conducted in accordance with the tenets of the Declaration of Helsinki and approved by the ethical committee of the University of York. Informed consent was sought from all participants prior to the experiment. The individual and processed data presented in this manuscript are available at https://osf.io/f2ta4/.

### Apparatus

Participants completed the experiment on the online platform Gorilla (Anwyl-Irvine, Massonnie, Flitton, Kirkham, & Evershed, 2020). At the beginning of the experiment, we estimated the screen size of each participant by using the “credit card” method (Li, Joo, Yeatman, & Reinecke, 2020). Participants were instructed to place a credit card (or any other card of the same format, with a width of 85.6 mm) against a credit card image on their screen and to adjust the image size using a slider (controlled by their mouse) so that the image matched the size of their actual card. We also estimated their viewing distance by asking them to report how far away from the screen they were sitting. We suggested a simple way to estimate this distance using their arm as reference: a picture of an arm was shown on the screen together with instructions informing them that, on average, the length of a forearm, measured from the elbow to the tip of the middle finger, is ∼43 to 48 cm. We stressed the importance of keeping the same distance from the screen throughout the experiment. We used these two estimates (i.e., screen size and viewing distance) to adjust the absolute size of our stimuli so that their size in degrees of visual angle was kept constant across participants.

### Stimuli and procedure

**Figure 1.**
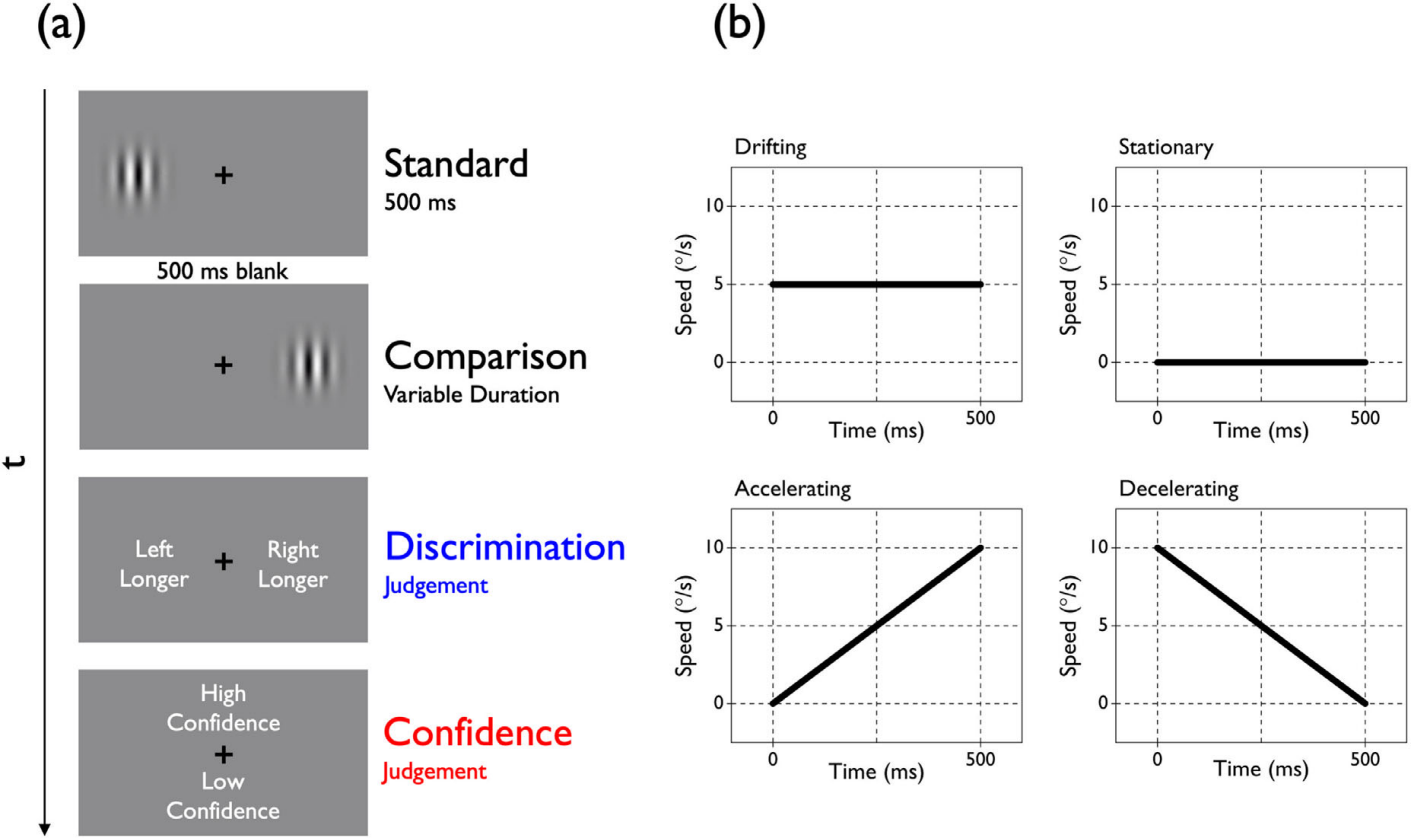
Experimental task and stimuli. (a) Schematic illustration of the procedure adopted in Experiments 1 and 2. Two visual stimuli (luminance-modulated Gabors similar to those depicted here in Experiment 1; simple gratings in Experiment 2) were sequentially presented (separated by a 500-ms blank page) and participants were required to make two decisions using their computer keyboard. First, they had to judge the relative duration of the intervals that contained the test stimuli (“Which is longer?”), and then they rated their confidence in their discrimination judgment (“How confident are you your response was correct?”). The standard duration was fixed across trials (500 ms), whereas the comparison duration varied on a trial-by-trial basis (between 200 and 800 ms in Experiment 1 and between 50 and 950 ms in Experiment 2). (b) Speed profiles over time for the standard stimulus in the four experimental conditions. In the drifting condition, the speed of the standard remained constant at 5°/s across the interval. In the stationary condition, there was no motion associated with the standard. In the accelerating condition, the speed of the standard increased linearly across the interval from 0°/s to 10°/s (average speed, 5°/s). In the decelerating condition, the speed of the standard decreased linearly across the interval from 10 to 0°/s (average speed, 5°/s). In all of the experimental conditions, the comparison stimulus always drifted at a constant rate of 5°/s.

Participants were required to fixate a black cross in the center of the screen while two test stimuli were sequentially displayed, separated by a 500-ms blank page (Figure 1a). One of the intervals, containing the standard stimulus, had the same duration across trials (500 ms), whereas the duration of the other interval, containing the comparison stimulus, varied on a trial-by-trial basis to generate a psychometric function. Both the presentation order (standard first or standard second) and the relative spatial location of the two test stimuli (standard left or standard right) were randomized across trials to avoid a response bias and visual adaptation, respectively. Participants were required to first report which interval appeared to have the longer duration (discrimination judgment) and then indicate whether their confidence in their judgment was either high or low (confidence judgment).

The comparison stimulus drifted at a constant speed of 5°/s, whereas we manipulated the speed profile of the standard stimulus across four experimental conditions (Figure 1b). In the drifting condition, the standard drifted at the same constant speed as the comparison; in the stationary condition, the standard was stationary; in the accelerating condition, the speed of the standard increased linearly across the interval between 0 and 10°/s (average speed: 5°/s); and, finally, in the decelerating condition, the speed of the standard decreased linearly across the interval between 10°/s and 0°/s (average speed, 5°/s). Standard and comparison always drifted in opposite left–right directions (except for the stationary condition, where no drifting was associated with the standard).

The stimuli were generated in Psychopy (Peirce et al., 2019), and each trial type was saved as an MP4 video file (H.264 format, 60 frames per second) and then uploaded in the experimental setup on Gorilla. A trial-type video contained the following sequence of events: initial blank page (500 ms), first test interval (standard or comparison), blank page between the tests (interstimulus interval = 500 ms), second test interval (standard or comparison), then final blank page (300 ms). For each participant and for each trial, we calculated the difference between the start and end times of the video playback in Gorilla and compared it with the expected trial duration to make sure that the timing of the video presentation was not substantially distorted (see next section).

### Exclusion criteria

For both experiments, we adopted four different types of exclusion criteria to make sure that the parameters we extracted from psychometric fits were of sufficient quality. First, participants were prevented from starting the online experiment unless they responded correctly to three comprehension checks regarding where they needed to look, what characteristic of the stimulus they needed to respond to, and what the confidence judgment referred to. Second, participants were excluded according to the following goodness-of-fit criteria (based on pilot data): if half of the confidence interval for either the duration point of subjective equality (PSE) or the point of minimal confidence (PMC) estimate was larger than 325 ms; if the *R*^2^ for either the duration PSE or the PMC estimate was lower than 0.15; and if the proportion of low confidence responses for both the shortest and the longest interval was larger than 0.5. Third, we ran an outlier analysis, and participants were excluded if the PSE, the PMC, the just noticeable difference (JND), or the full width at half height (FWHH) differed by more than three scaled median absolute deviations (MADs) from the group median in any given condition. Finally, participants were excluded if the mean difference between the mean duration of the video playback in Gorilla (averaged across all trials) and the actual mean video duration (averaged across all video durations) exceeded ±10% of the actual mean video duration (which was 2300 ms for both Experiments 1 and 2).

For Experiment 1, out of 84 participants that were initially recruited, we included in the data analysis 33 participants (24 identifying as female, nine as male; mean age ± *SD*, 31.63 ± 12.33 years; mean playback error [video playback duration – actual video duration], −8.61 ± 41.06 ms). Experiment 2 included 53 participants out of 106 initially recruited (35 identifying as female, 18 as male; mean age, 29.92 ± 8.09 years; mean playback error, −19.69 ± 17.72 ms).

To our knowledge, Experiment 1 was the first attempt at measuring confidence judgments in an online study on time perception. Therefore, the sample size we deemed acceptable for Experiment 1 was based on previous psychophysical and neuroimaging studies on the effects of stimulus speed on time perception (Binetti et al., 2012; Binetti et al., 2020; Bruno et al., 2015; Matthews, 2011; Sasaki et al., 2013), where the sample size varied between 5 and 28 participants. We decided to opt for a larger sample size as we expected higher inter-individual variability with data collected online, under less controlled conditions. For Experiment 2, we ran a power analysis using G*Power 3.1 (Faul, Erdfelder, Buchner, & Lang, 2009). We calculated that, for a repeated-measures analysis of variance (ANOVA), we needed 53 participants to be able to detect a small to medium effect size (*F* = 0.2) with an alpha error probability of 0.05 and a power of 0.8. Our estimate of the correlation between repeated measures was based on what we observed in Experiment 1 (average Pearson's *r* = 0.25).

### Pre-registration

Experiment 2 was pre-registered (https://osf.io/59ywt). The following statistical analyses were exploratory and we did not pre-register them: one-way ANOVAs on precision estimates and confidence peak heights, Deming regression analysis, and all of the paired-samples *t*-tests. The description of the model was not pre-registered, either.

## Experiment 1: Stimulus speed affects both duration discrimination and confidence judgments

### Methods

In Experiment 1, participants had to pay attention to the relative duration of two subsecond intervals containing luminance-modulated Gabor gratings (vertically oriented, spatial frequency = 1 c/°), displayed 5° away from the center of the monitor on the horizontal midline. The diameter of the grating window was 5° of visual angle, the standard deviation of the Gaussian spatial envelope was 0.83° of visual angle and the Michelson contrast was 100%. The standard had a fixed duration (500 ms) across trials and a variable speed profile across conditions. The comparison interval always contained a drifting grating (constant speed of 5°/s), whereas its duration varied on a trial-by-trial basis in seven steps (200, 300, 400, 500, 600, 700, or 800 ms). At the end of each trial, participants had to report, first, a discrimination judgment (“Which was the longer interval?”) and then a confidence judgment (“How confident are you your duration judgment was correct?”). Psychometric fits were determined for each participant.

In Experiment 1, the combination of speed profiles, interval durations, relative locations, and presentation orders yielded a total of 112 different trial types (4 experimental conditions × 7 durations × 2 standard/comparison relative locations × 2 presentation orders). Each trial was saved as an MP4 video file (H.264 format, 60 frames per second) and then uploaded in the experimental setup on Gorilla. Participants completed an initial block of 28 practice trials (one repetition for each of the seven comparison durations for each of the four experimental conditions) to familiarize themselves with the task, followed by four experimental blocks (one for each experimental condition) of 140 trials each (corresponding to 20 repetitions for each of the seven comparison durations presented in a randomized order), for a total of 560 experimental trials. The order of the blocks was randomized across participants.

### Data analysis

We fitted cumulative Gaussian functions through the individual and mean discrimination data. They described the proportion of trials where the interval containing the comparison was judged to be longer than the interval containing the standard, as a function of the comparison duration. The PSE (defined as the 50% point on the fitting function) was our discrimination measure of perceived duration. The JND, corresponding to half the distance between the 25% and 75% points on the psychometric function, was our measure of the precision of participants’ discrimination estimates.

We fitted raised Gaussian functions through the individual and mean confidence data, which described the proportion of “low confidence” responses as a function of the comparison duration. The peak center of the Gaussian fit, which is the PMC, was our confidence measure of perceived duration. The FWHH of the Gaussian fit was our measure of the precision of participants’ confidence estimates.

**Figure 2.**
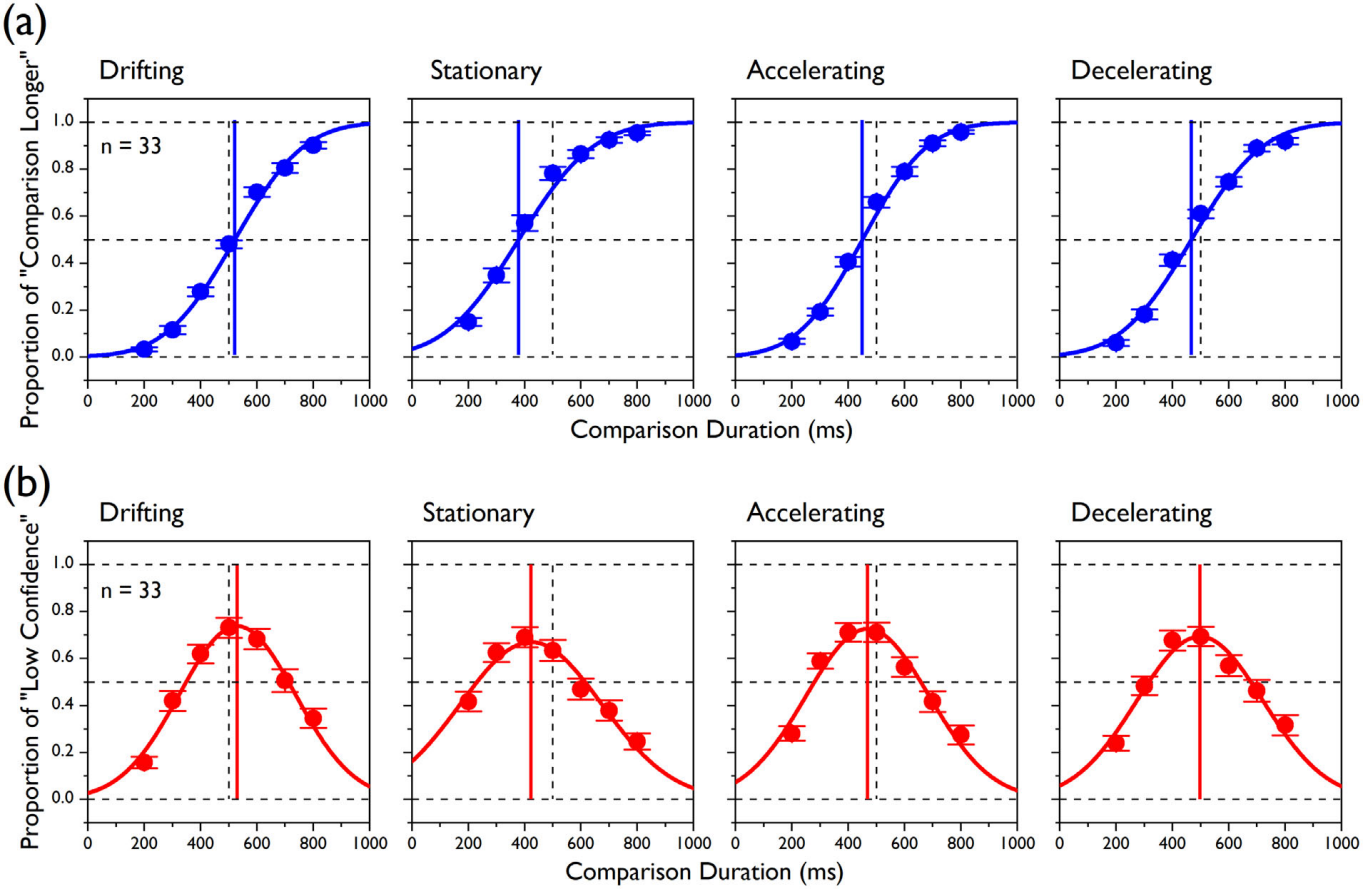
Group data in Experiment 1. (a) Cumulative Gaussian functions (fitted through data from all participants at once; blue fits) are plotted for discrimination judgments together with the mean proportions of “comparison longer” responses (averaged across all participants; blue circles) for all the seven comparison durations and for the four experimental conditions. Note that those fits are shown here and in panel b for reference but the statistics are based on psychometric fits to individual data. The vertical blue lines represent the PSEs (i.e., corresponding to the 50% points on the functions), and the vertical dashed lines (here and in panel b) indicate the actual standard duration (i.e., 500 ms). (b) Raised Gaussian functions (red fits) are plotted for confidence judgments together with the mean proportions of “low confidence” responses for all of the comparison durations and all of the experimental conditions. The vertical red lines point to the duration levels corresponding to the PMCs (i.e., the peaks of “low confidence” responses). Error bars indicate ±1 *SEM*.

All of the duration and precision estimates used for statistical analyses were derived from individual fits. For reference, we provide the fitted functions based on the data of all participants in Figures 2 and 4.

We ran a repeated-measures ANOVA (4 × 2) to test the effect of stimulus type (stationary, drifting, decelerating, or accelerating), judgment (discrimination or confidence), and their interaction on our central tendency (i.e., PSE and PMC) measures. As we observed a small but consistent bias in the drifting condition (which we considered our baseline condition as there was no difference between the speed profiles of the standard and comparison intervals), we also tested the same effects on the changes in PSE and PMC relative to the same measures in the drifting condition. In this case, the stimulus type factor had three levels, corresponding to the perceived duration change for the stationary, accelerating, and decelerating conditions. Where required, we applied the Greenhouse–Geisser correction for violation of sphericity. Significant effects of stimulus type on the measures of central tendency were followed up by planned contrasts testing that stationary < accelerating < decelerating < drifting (i.e., three tests) for each judgment type. The Bonferroni correction for multiple comparisons was applied to the significance level of the planned contrasts (*p* = 0.05/3 = 0.0167) and of the paired-sample *t*-tests between the PSEs and the PMCs for each of the four speed conditions (*p* = 0.05/4 = 0.0125), as well as those between the PSE and PMC changes in the stationary, accelerating, and decelerating conditions relative to the drifting condition (*p* = 0.05/3 = 0.0167). We ran separate one-way ANOVAs to test the effect of stimulus type on our precision measures (i.e., JND and FWHH) and on the peak heights of the confidence functions (i.e., curve height at PMC). The *F* statistic of the Brown–Forsythe test was reported when the assumption of homogeneity of variances was violated.

### Results

In Figure 2, we plotted the mean psychometric fits and data points (averaged across all participants) for the two judgment types (i.e., discrimination and confidence) and for the four speed profiles of the standard stimulus (i.e., drifting, stationary, accelerating, and decelerating). As far as the discrimination judgments are concerned (Figure 2a), the mean psychometric functions appeared steep and ordered, and the errors associated with the mean data points are small. The mean data points for the confidence judgments (Figure 2b) were well captured by raised Gaussian functions. As expected, the degree of uncertainty was associated with the duration difference between the standard and comparison interval, so that smaller duration differences yielded a higher proportion of low confidence judgments.

It is noticeable that even the highest proportion of “low confidence” responses (i.e., the curve height at the point of minimal confidence) for all speed profiles was substantially smaller than 1, suggesting that participants were generally overconfident about the correctness of their duration judgments even when there was no actual difference between the standard and comparison durations. On the other hand, near the tails of the distribution, when the comparison duration was substantially different from that of the standard, the participants’ uncertainty was above 0, indicating a lack of confidence, especially for very long comparison durations.

**Figure 3.**
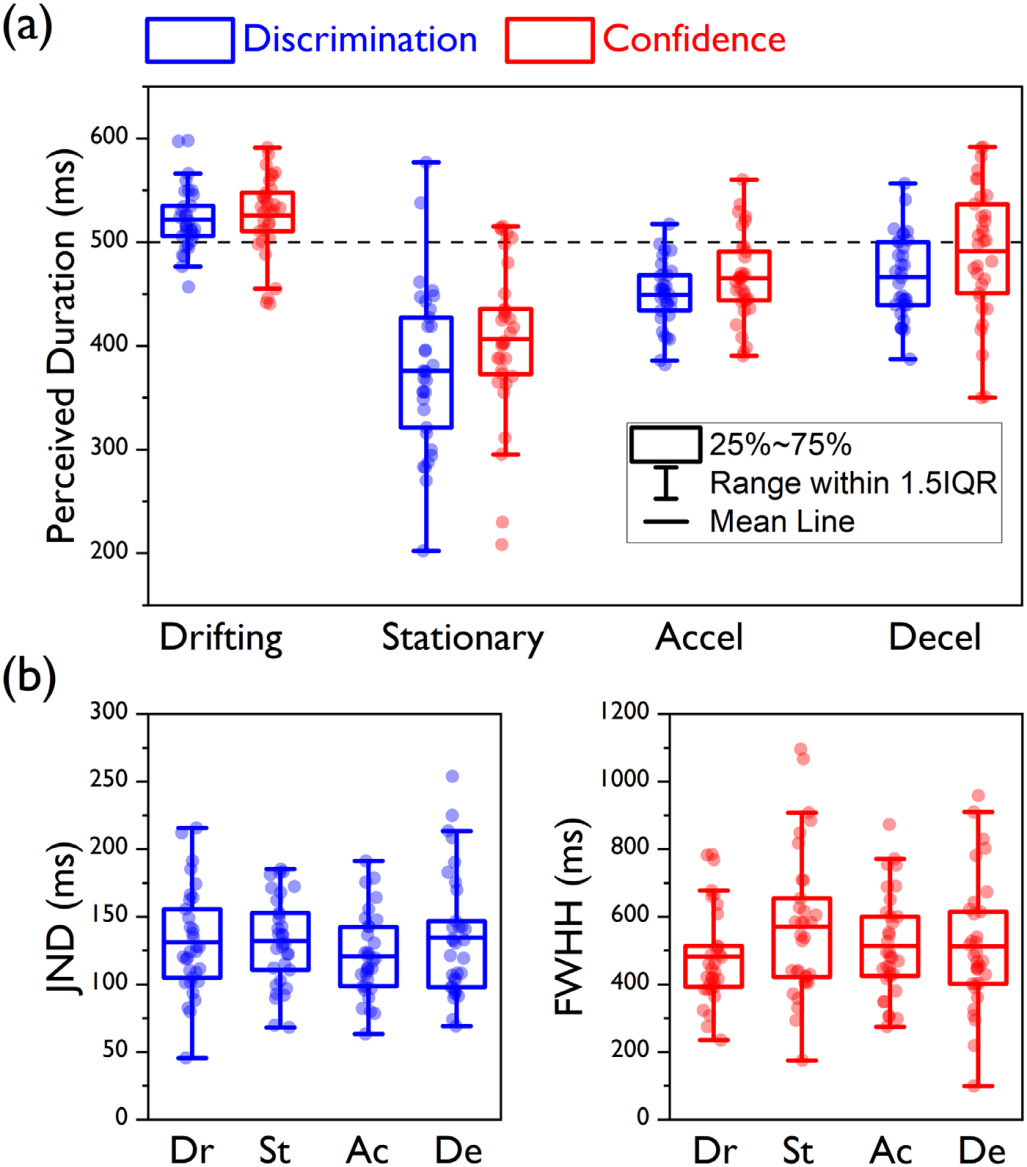
Central tendency and precision estimates for Experiment 1. (a) Box plots for the PSEs (blue) and the PMCs (red) are presented here for all of the experimental conditions. Here and in panel b, the boxes are drawn from the first to the third quartile, the blue and red horizontal lines depict the means, and the whiskers are drawn to the highest and lowest data points within 1.5 times the distance between the first and third quartile (i.e., the interquartile range) from above the third quartile and from below the first quartile, respectively. Circular symbols represent individual PSE and PMC estimates. The horizontal dashed line indicates the actual standard duration. (b) Box plots for the JNDs of the discrimination judgments (blue, left panel) and the FWHHs for the confidence judgments (red, right panel) for all of the experimental conditions.

In this study, we were mainly interested in the degree of alignment between the PSE, which was our discrimination measure of perceived duration, and the PMC, which was our confidence measure of perceived duration. Figure 2 shows that there was a fair degree of alignment between the two measures of central tendency. We used the individually determined estimates to analyze this effect statistically (individual and mean estimates for PSE and PMC are plotted in Figure 3a).

The speed profile of the standard stimulus affected its perceived duration; for the main effect for speed condition, *F*(1.916, 61.328) = 60.01, *p* < 0.0001. As expected, the analysis of the PSEs showed that the largest duration compression (∼25% reduction relative to the actual standard duration, 500 ms) was observed when the standard interval contained a stationary stimulus (mean PSE ± *SD*, 376.18 ± 77.93 ms), whereas the bias was equally smaller for the accelerating (449.38 ± 31.34 ms) and decelerating (466.33 ± 39.62 ms) conditions (Bonferroni-corrected *p* = 0.0167). For planned contrasts: decelerating < drifting, *t*(60.41) = 6.34, *p* < 0.0001; accelerating < decelerating, *t*(60.77) = 1.93, *p* = 0.059; and stationary < accelerating, *t*(42.09) = 5.01, *p* < 0.0001.

A significant main effect for judgment type, *F*(1, 32) = 8.001, *p* = 0.008, indicated that, overall, the mean PMCs were shifted toward higher values relative to the PSEs. This difference was not significantly different across the four speed profiles (interaction speed condition × judgment type), *F*(1.83, 58.67) = 2.04, *p* = 0.143. However, the mean PMCs were modulated by the speed profile in the same fashion as the mean PSEs: The peaks were associated with the shortest durations in the stationary condition (mean PMC, 406.53 ± 74.21 ms), whereas those in the accelerating condition (465.35 ± 41.47 ms) and decelerating condition (491.13 ± 63.54 ms) were associated with longer durations than in the stationary condition but shorter than in the drifting condition (Bonferroni-corrected *p* = 0.0167). For planned contrasts: decelerating < drifting, *t*(52.93) = 2.67, *p* = 0.01; accelerating < decelerating, *t*(55.07) = 1.95, *p* = 0.056; and stationary < accelerating, *t*(50.21) = 3.97, *p* < 0.0001. Also, none of the comparisons between PSE and PMC across conditions turned out to be statistically significant after correcting for multiple comparisons (Bonferroni-corrected *p* = 0.0124). For paired-samples *t*-tests: drifting, *t*(32) = 0.51, *p* = 0.613; stationary, *t*(32) = 2.57, *p* = 0.015; accelerating: *t*(32) = 2.13, *p* = 0.041; and decelerating, *t*(32) = 2.30, *p* = 0.028.

The same pattern of results emerged when the drifting condition was used as a baseline. By subtracting the PSE and PMC of the drifting condition from those of the other speed conditions for each participant and then testing the effects of speed condition and judgment type, we once again observed significant main effects for both speed condition, *F*(1.65, 52.92) = 33.92, *p* < 0.0001, and judgment type, *F*(1, 32) = 8.01, *p* = 0.008, but no significant interaction, *F*(1.5, 47.89) = 0.664, *p* = 0.478. Also, none of the comparisons between PSE and PMC changes relative to the drifting condition reached statistical significance after correcting for multiple comparisons (Bonferroni-corrected *p* = 0.0167. For paired-samples *t*-tests: stationary duration change, *t*(32) = 1.95, *p* = 0.06; accelerating duration change, *t*(32) = 1.65, *p* = 0.108; and decelerating duration change, *t*(32) = 2.52, *p* = 0.017.

The JNDs extracted from the psychometric functions for the discrimination judgments (Figure 3B, blue symbols and box plots) were not different across conditions, *F*(3, 128) = 0.86, *p* = 0.466. Similarly, no difference was detected for the FWHHs for the confidence judgments (Figure 3B, red symbols and box plots), *F*(3, 128) = 1.42, *p* = 0.239, indicating that the different speed profiles of our stimuli had comparable effects on the precision of both the discrimination and confidence judgments. The proportion of “low confidence” responses peaked at values that were substantially smaller than 1 (mean peak heights: drifting, 0.75 ± 2.46; stationary, 0.71 ± 0.21; accelerating, 0.75± 0.21; decelerating, 0.74 ± 0.21), implying that participants were generally overconfident even when their performance was at chance. This tendency was not influenced by the speed profile of the test stimuli, *F*(3, 128) = 0.21, *p* = 0.891.

## Experiment 2: Participants did not use feedback to calibrate their confidence

To help our participants to better calibrate their confidence judgments, in Experiment 2 we let participants know if their duration judgment was correct at the end of each of the 32 practice trials. We also used a wider range of durations for the comparison interval, adding anchor points (i.e., durations that were very clearly shorter or longer than 500 ms), and we interleaved trials from the different speed conditions within the same block (rather than having all the trials from one condition in the same block, as in Experiment 1).

We deemed these latter changes necessary, as in the drifting condition of Experiment 1, where no change in perceived duration was expected (as both standard and comparison had the same speed profile), we actually observed some small but significant duration dilation for both the discrimination, with mean PSE = 521.78 ± 30.89 and one-sample *t*-test against 500, *t*(32) = 4.05, *p* < 0.001, and confidence judgments, with mean PMC = 525.72 ± 38.78, *t*(32) = 3.81, and *p* = 0.001. We thought this unexpected bias might have been due to two factors: first, the blocked presentation, and, second, the narrow duration range. If we look at the panel corresponding to the drifting condition in Figure 2a, we can see that, when standard and comparison intervals had the same duration, participants were at chance, as expected, but they underestimated the duration of the longer intervals (especially 800 ms), and this shifted the PSE toward higher values.

### Methods

The overall structure of Experiment 2 was identical to that of Experiment 1, with the following exceptions. The stimuli were not Gabors but simple luminance-modulated gratings. The duration of the comparison stimulus varied across trials in nine steps (50, 162, 275, 388, 500, 612, 725, 838, and 950 ms). Participants completed five experimental blocks of 144 trials each, for a total of 720 experimental trials (4 experimental conditions × 9 durations × 2 standard/comparison relative locations × 2 presentation orders × 5 repetitions). We interleaved trials from the different conditions within the same block. The initial practice block consisted of 32 trials (four repetitions for each of the eight comparison durations—we did not include trials where standard and comparison had the same duration—from the drifting condition only), and, at the end of each practice trial (which required a duration judgment only), participants received feedback about their performance. No feedback was provided for experimental trials.

### Data analysis

After conducting the same statistical analyses as in Experiment 1, the higher number of participants and comparison durations in Experiment 2 also allowed us to further explore our data by performing an orthogonal (or Deming) regression (Deming, 1943; Hall, 2014; Kane & Mroch, 2020) between our discrimination and confidence estimates of perceived duration for each speed condition. This analysis can be used to determine the equivalence of measurement instruments. Unlike linear regression, orthogonal regression assumes that both the dependent and the independent variables (which are supposed to be linearly correlated) are measured with error (as is the case in the present study), and it minimizes the distances of the data points in both the *x* and *y* directions from the fitted line; that is, it minimizes the sum-of-squared orthogonal deviations. It also produces confidence interval estimates for the slope and the intercept of the orthogonal fit, which can be used to test whether the two parameters are significantly different from 1 and 0, respectively, indicating a deviation from a perfect linear correlation between the two measures. In addition, we determined the Bayes factor, which gave us the amount of evidence favoring the reduced model (with slope fixed to 1 and intercept fixed to 0) over the orthogonal model given the data. To calculate the Bayes factor, we used the large sample approximation method (Burnham & Anderson, 2004). A similar application of this method can be found, for example, in Schütz, Kerzel, and Souto (2014). We first determined the Bayesian information criterion (BIC) (Schwarz, 1978) for both methods:

$$\begin{array}{c} \mathrm{BIC}=n\ln\left(\frac{RSS}{n}\right)+k\ln\left(n\right) \end{array}$$

where *n* corresponds to the number of participants, *RSS* is the residual sum of squares, and *k* is the number of free parameters (0 for the reduced model and 2 for the orthogonal model). Then, for each model *i*, we determined the posterior probability *p*:

$$\begin{array}{c} p_{i}=\;\frac{e^{-0.5\Delta BIC_{i}}}{\sum_{r=1}^{R}e^{-0.5\Delta BIC_{r}}} \end{array}$$

where ∆*BIC* is the difference, for each model, between the *BIC* for that model and the lower *BIC* between the two models (i.e., the ∆*BIC* for the minimum *BIC* model is 0). Finally, the Bayes factor was calculated as the ratio between the two posterior probabilities:

$$\begin{array}{c} BF_{10}=\;\frac{p_{reduced}}{p_{orthogonal}} \end{array}$$

### Results

**Figure 4.**
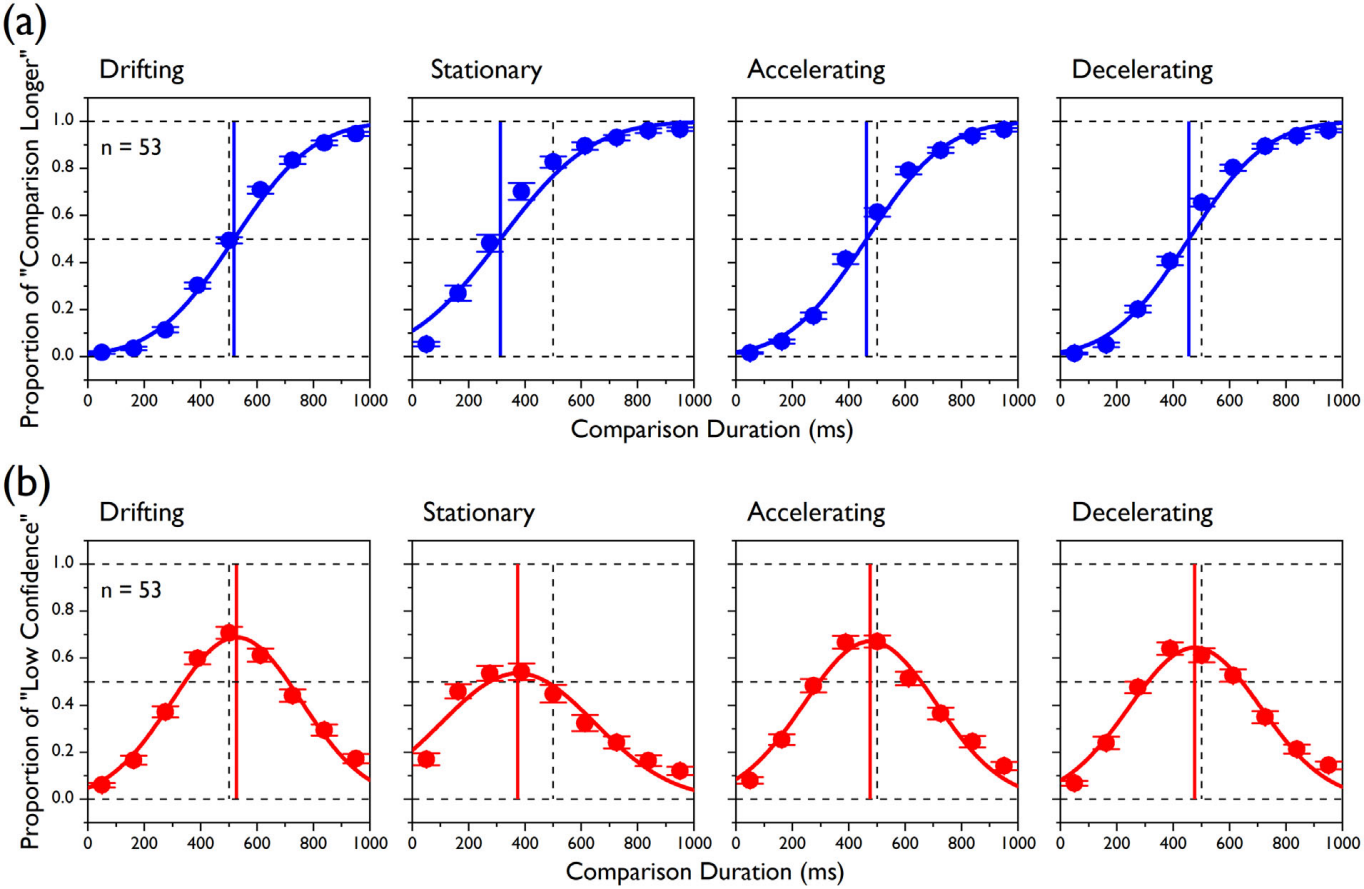
Group data in Experiment 2. (a) Cumulative Gaussian functions (fitted through data from all participants at once; blue fits) are plotted for discrimination judgments together with the mean proportions of “comparison longer” responses (averaged across all participants; blue circles) for all of the nine comparison durations and for the four experimental conditions. Note that those fits are shown here and in panel b for reference, but the statistics are based on psychometric fits to individual data. The vertical blue lines represent the PSEs (i.e., corresponding to the 50% points on the functions), and the vertical dashed lines (here and in panel b) indicate the actual standard duration (i.e., 500 ms). (b) Raised Gaussian functions (red fits) are plotted for confidence judgments together with the mean proportions of “low confidence” responses for all of the comparison durations and all of the experimental conditions. The vertical red lines represent the PMCs (corresponding to the peaks of “low confidence” responses). Error bars indicate ±1 *SEM*.

Figure 4 shows the mean psychometric functions for both the judgment types and for all the speed conditions of Experiment 2. As in Experiment 1, the functions for the discrimination judgments were steep and ordered, and the confidence in the correctness of participants’ decision, as predicted by a raised Gaussian function, was determined by the magnitude of the difference between standard and comparison durations.

**Figure 5.**
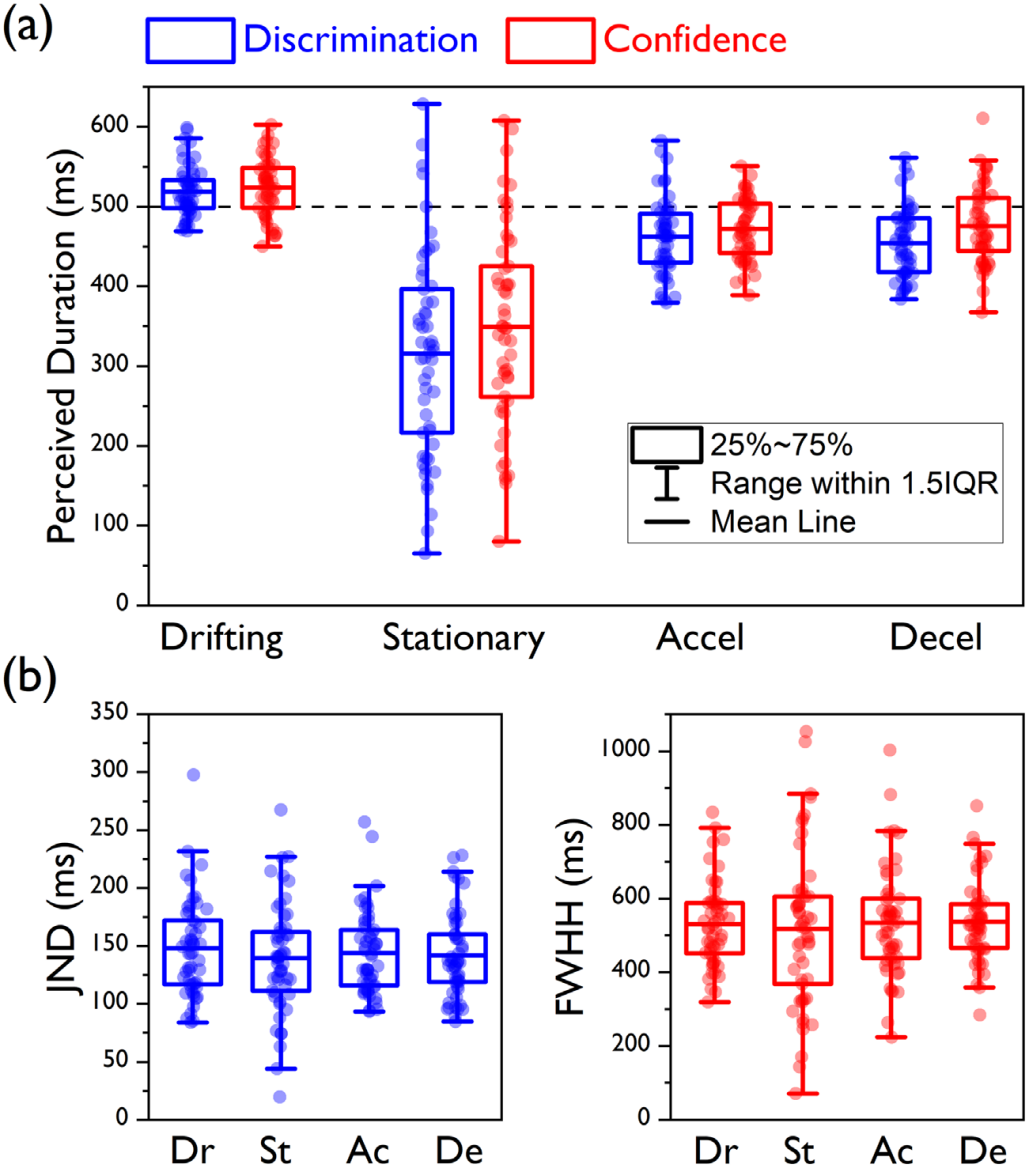
Central tendency and precision estimates for Experiment 2. (a) Box plots for the PSEs (blue) and the PMCs (red) are presented here for all of the experimental conditions. Here and in panel b the boxes are drawn from the first to the third quartile, the blue and red horizontal lines depict the means, and the whiskers are drawn to the highest and lowest data points within 1.5 times the distance between the first and third quartile (i.e., the interquartile range) from above the third quartile and from below the first quartile, respectively. Circular symbols represent individual PSE and PMC estimates. The horizontal dashed line indicates the actual standard duration. (b) Box plots for the JNDs of the discrimination judgments (blue, left panel) and the FWHH for the confidence judgments (red, right panel) for all of the experimental conditions.

We observed that the PSEs and the PMCs appeared to be modulated by the speed profile of the standard stimulus in a similar fashion, as confirmed by a significant main effect for speed condition (Figure 5a), *F*(1.38, 71.71) = 74.34, *p* < 0.0001, with some slight differences in the amount of shift predicted by the two measures. Perceived duration estimates provided by the PSEs revealed a very strong compression (∼35%) for the stationary standard (mean PSE, 315.67 ± 127.94), whereas milder compression was observed for accelerating standard (462.18 ± 47.1) and the decelerating standard (454.44 ± 42.71; Bonferroni-corrected *p* = 0.0167). For planned contrasts: decelerating < drifting, *t*(95.94) = 8.81, *p* < 0.0001; accelerating < decelerating, *t*(103.02) = –0.89, *p* = 0.377; and stationary < accelerating, *t*(65.84) = 7.82, *p* < 0.0001.

Perceived duration estimates provided by the PMCs were on average higher than for the PSEs—for the main effect of judgment type, *F*(1, 52) = 38.59, *p* < 0.0001—and the magnitude of this difference varied across conditions (interaction speed condition × judgment type), *F*(3,16) = 5.26, *p* = 0.002. In fact, although, as for the PSEs, the amount of duration compression estimated by the PMCs was maximal in the stationary condition (mean PMC, 349.51 ± 124.19) and equally milder in the accelerating condition (472.4 ± 38.11) and decelerating condition (475.59 ± 47.92; Bonferroni-corrected *p* = 0.0167). For planned contrasts: decelerating < drifting, *t*(96.78) = 5.82, *p* < 0.0001; accelerating < decelerating, *t*(98.99) = 0.38, *p* = 0.706; and stationary < accelerating, *t*(61.71) = 6.89, *p* < 0.0001. Comparisons between the two measures were significant only in the stationary and decelerating conditions after correcting for multiple comparisons (Bonferroni-corrected *p* = 0.0125). For paired-samples *t*-tests, drifting, *t*(52) = 0.99, *p* = 0.327; stationary, *t*(52) = 5.35, *p* < 0.0001; accelerating, *t*(52) = 1.72, *p* = 0.092; and decelerating, *t*(52) = 4.09, *p* < 0.0001.

As for Experiment 1, this pattern of results remained unchanged when we ran the same analyses on the differences in PSE and PMC relative to the drifting condition. We observed significant main effects for speed condition, *F*(1.24, 64.41) = 54.84, *p* < 0.0001, and judgment type, *F*(1, 52) = 9.54, *p* = 0.003, as well as for the interaction between these two factors, *F*(2, 104) = 3.91, *p* = 0.023. Only for the stationary condition did the comparison between PSE and PMC changes reach statistical significance after correcting for multiple comparisons (Bonferroni-corrected *p* = 0.0167. For paired-samples *t*-tests, stationary duration change, *t*(52) = 3.73, *p* < 0.0001; accelerating duration change, *t*(52) = 0.73, *p* = 0.472; and decelerating duration change, *t*(52) = 2.43, *p* = 0.019.

**Figure 6.**
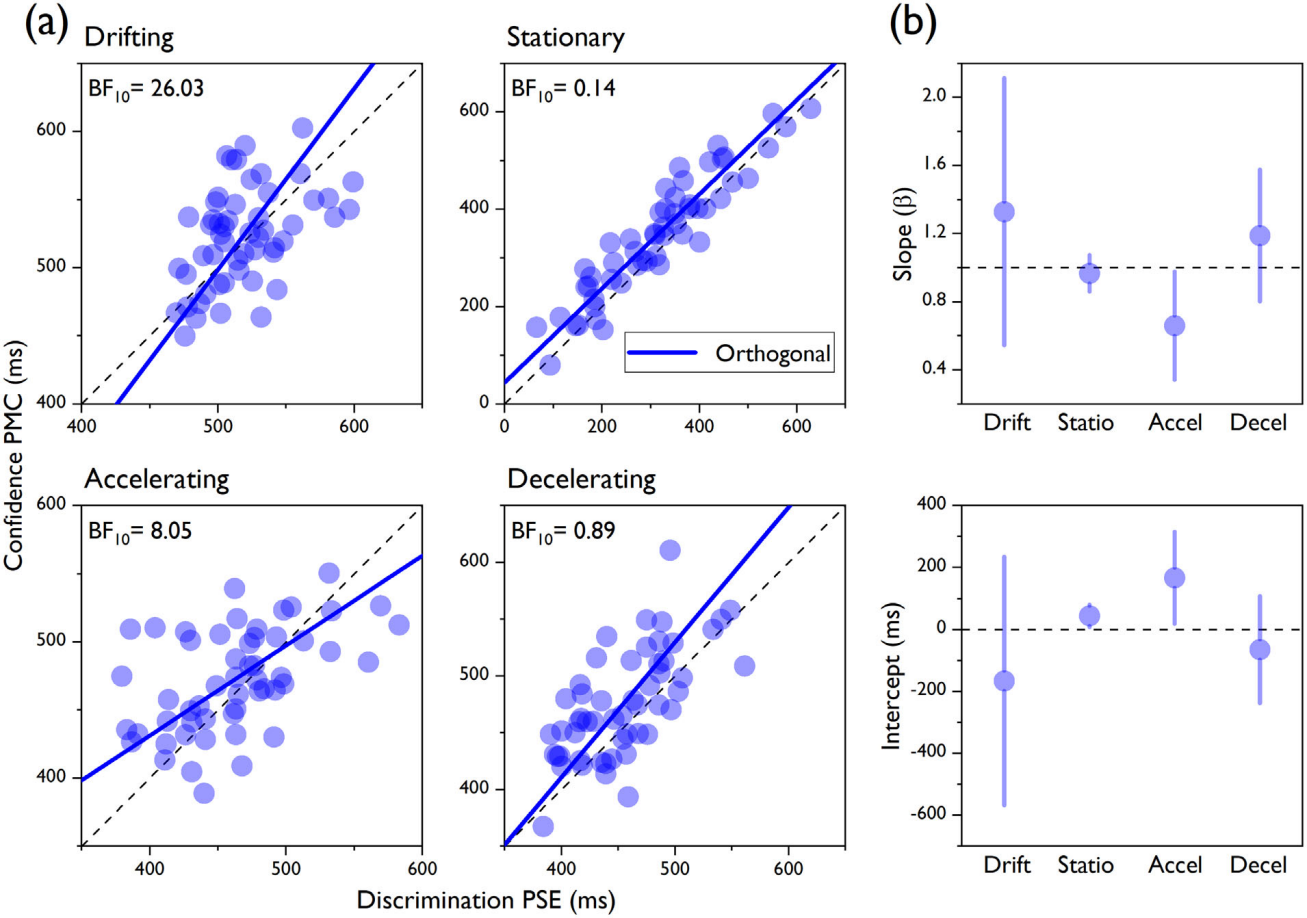
Orthogonal regressions. (a) The individual PMCs are plotted on the *y*-axis and the individual PSEs are plotted on the *x*-axis for all of the experimental conditions of Experiment 2. Orthogonal regression lines are depicted in blue. The Bayes factors are reported. The dashed lines represent the equality lines (*y* = *x*). (b) Slopes (top panel) and intercepts (bottom panel) for the orthogonal regression lines are plotted in blue for all of the speed conditions. Error bars describe 95% confidence intervals.

To gain a better understanding of the underlying functional relationship between our two central tendency estimates, we conducted an orthogonal or Deming regression analysis (Deming, 1943; Hall, 2014; Kane & Mroch, 2020), which can be simply thought of as a linear regression between two dependent variables (see Data analysis section). As orthogonal regression assumes that the two variables are linearly correlated, we first made sure this was the case for all of the speed conditions (all Pearson's *r* > 0.45, all *p* < 0.0001). The orthogonal fits (Figure 6a, blue lines) showed positive correlations that are not perfect. In fact, the 95% confidence intervals derived from the orthogonal regression (Figure 6b) crossed both the 1 line for the slope and the 0 line for the intercept only for the drifting and decelerating conditions. Furthermore, we determined the Bayes factor, which provides the amount of evidence supporting the null hypothesis, which here indicated that the data could be better fitted by a reduced model with fixed slope = 1 and fixed intercept = 0 (indicating a perfect correlation between the two estimates), over the alternative hypothesis that an orthogonal model with free-to-vary slope and intercept should be favored. A Bayes factor analysis provided strong and moderate evidence for the null hypothesis for the drifting (BF_10_ = 26.03) and accelerating (BF_10_ = 8.05) conditions, respectively, indicating that in those conditions the equality line was the best fitting model. However, there was moderate and anecdotal evidence favoring the alternative hypothesis for the stationary (BF_10_ = 0.137) and decelerating (BF_10_ = 0.8857) conditions, respectively, implying that the two estimates were not perfectly correlated.

As in Experiment 1, both the JNDs (Figure 5b) for the discrimination judgments, *F*(3, 208) = 0.41, *p* = 0.742, and the FWHHs for the confidence judgments, *F*(3, 155.4) = 0.17, *p* = 0.918, did not differ across conditions. Also, the feedback during training did not improve the calibration of participants’ confidence, as the peak heights were still substantially smaller than 1 (mean peak heights: drifting, 0.71 ± 1.79; stationary, 0.64 ± 0.19; accelerating, 0.7± 0.18; decelerating, 0.67 ± 0.19), indicating overconfidence when their performance was at chance. The amount of overconfidence did not change across speed conditions, *F*(3, 208) = 1.41, *p* = 0.242.

## Modeling: A simple observer model captures inter-individual differences in the confidence criterion

We designed an observer model to explain what factors influenced the precision of discrimination and confidence judgments. The model assumed that both discrimination and confidence judgments were based on duration discriminability (individually estimated as the JND based on the actual discrimination judgment). It then simply compared the duration signals associated with the standard and comparison intervals to decide which was longer. For confidence judgments, the difference between the two duration signals divided by our discriminability measure had to exceed an internal confidence criterion for the model to report high confidence.

### Methods

We created a simulated dataset consisting of the same number of participants as in Experiment 2. For each judgment type and speed condition, the model generated a psychometric function using the same number of trials used in Experiment 2. For each trial, the model generated a simulated duration signal (SDS), the value of which was randomly sampled from a normal distribution with the actual duration as the mean and the real JND (extracted from the function of the real participant whose discrimination and confidence the model aimed to predict) as the standard deviation:

$$\begin{array}{c} SDS\;∼\;N(Actual\;Duration,\;JND_{real}) \end{array}$$

The model predicted a “comparison longer” judgment if the SDS for the comparison duration exceeded that for the standard duration:

$$SDS_{Comp}>SDS_{Stand}$$

The model predicted a “high confidence” judgment if the ratio of the absolute difference between the SDSs for the two tests to the real JND exceeded the confidence criterion:

$$\frac{\left|SDS_{Comp}-\;SDS_{Stand}\right|}{JND_{Real}}>Confidence\;Criterion$$

The confidence criterion was the only free parameter of our model, and for each simulated participant we chose the value that minimized the absolute difference between the FWHH of the simulated confidence curve and that of the real confidence curve. After having determined the criterion for each participant, we extracted the JNDs for the discrimination judgments and the FWHHs for the confidence judgments from the simulated dataset. For each speed condition, we then ran a linear regression analysis to test how well the simulated JNDs and FWHHs could predict the real ones. BICs were calculated as described above for a linear model with the slope and intercept free to vary and for a reduced model with fixed slope = 1 and fixed intercept = 0; the Bayes factor was determined for each speed condition.

### Results

**Figure 7.**
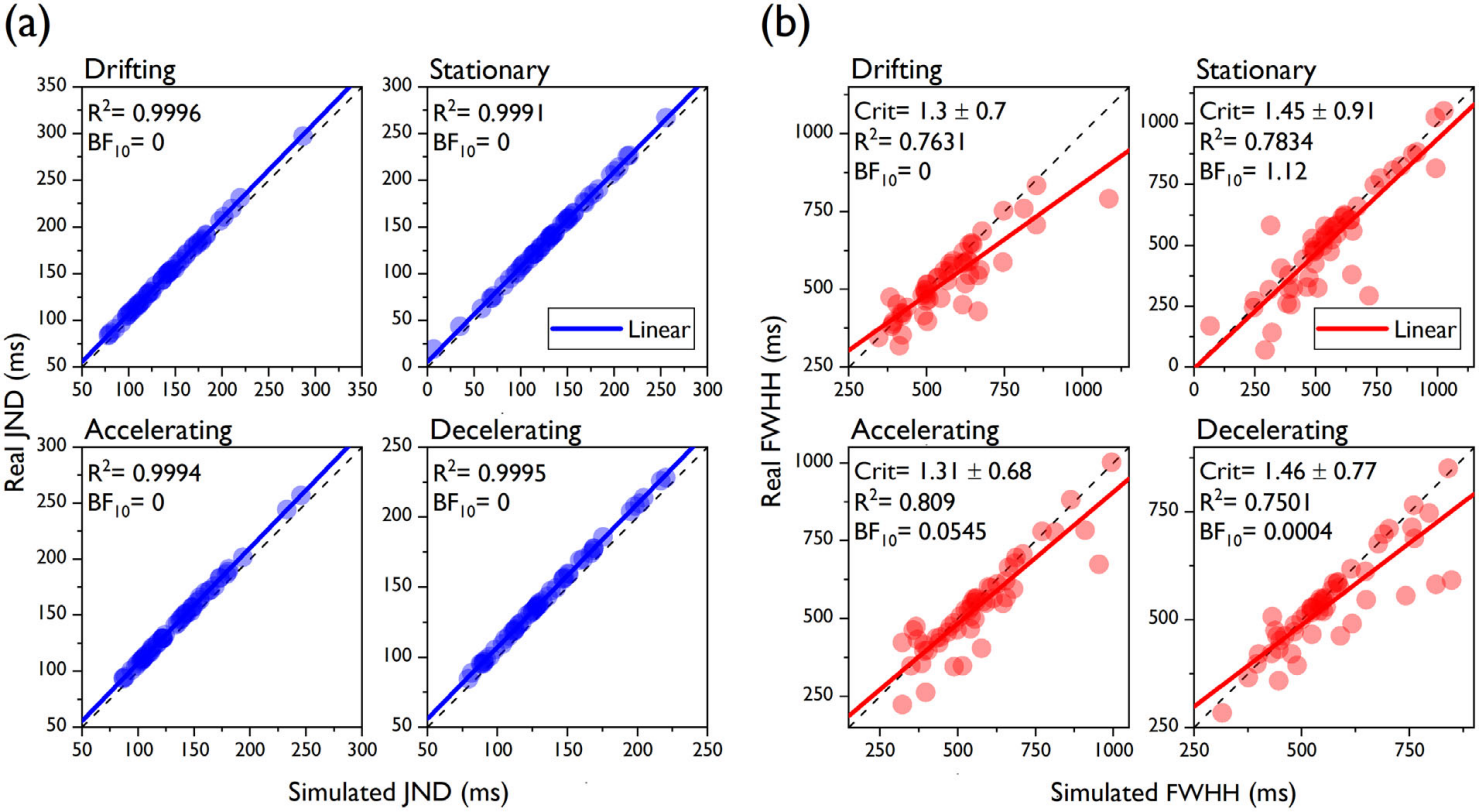
Simulated vs. real precision estimates. (a) The real individual JNDs of the discrimination judgments from Experiment 2 are plotted in blue as a function of the simulated JNDs, which were generated by an observer model (see main text). Linear regression lines are represented in blue. The *R*^2^ and the Bayes factors are also reported here and in panel b. (b) The real individual FWWHs of the confidence judgments are plotted in red against the simulated FWWHs generated by the same observer model as in panel a. Linear regression lines are represented in red. The mean confidence criterions (averaged across all participants ± 1*SD*) are also reported.

For the discrimination judgments (Figure 7a), virtually all of the variance in the real data was predicted by the simulation (all *R*^2^ > 0.9991). For all of the speed conditions, the Bayes factor was 0, indicating extremely large evidence against the reduced model. For the confidence judgments, between 75% and 80% of the variance in the real data was captured by the simulation. Bayes factors revealed anecdotal evidence supporting the reduced model in the stationary condition (BF_10_ = 1.12), whereas it provided strong to very strong evidence in favor of the alternative model in the accelerating (BF_10_ = 0.0545) drifting (BF_10_ = 0) and decelerating (BF_10_ = 0.0004) conditions. The mean confidence criterion estimate ranged from 1.3 to 1.46 across speed conditions, indicating that, on average, the difference between the comparison and standard duration signals had to be almost 1.5 times as big as the JND for our participants to report high confidence in their decision.

**Figure 8.**
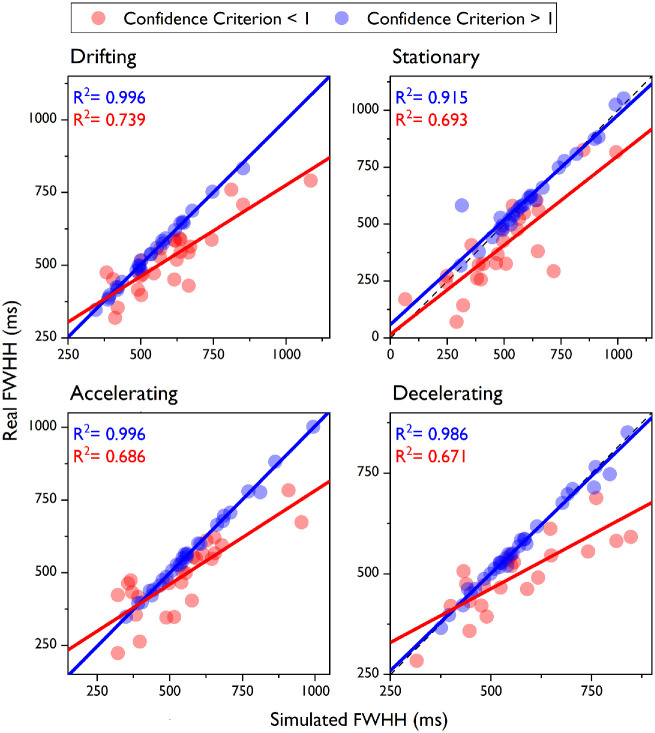
Inter-individual differences. We show here the performance of our observer model for different confidence criterion values. The real FWHHs are plotted as a function of the FWHHs predicted by our model. Data are the same as in Figure 7b, but they were split into two groups: participants with a confidence criterion lower (red symbols) or higher (blue symbols) than 1. Linear regression lines are plotted in red and blue. The *R*^2^ values for all of the speed conditions are also reported.

When we looked more closely at individual differences in the confidence criterion estimates, a clear pattern emerged. Figure 8 shows that the performance of our model in minimizing the absolute difference between real and simulated FWHHs was substantially better when the confidence criterion estimate was higher than 1. A linear regression analysis conducted on the two criterion ranges separately revealed that almost all of the variance in the real data was predicted by the model for participants with criterion > 1 (*R*^2^ values: drifting, 0.996; stationary, 0.915; accelerating, 0.996; decelerating, 0.986), whereas about 70% of the variance was explained for those with criterion < 1 (*R*^2^ values: drifting, 0.739; stationary, 0.693; accelerating, 0.686; decelerating, 0.671). A criterion of 1 or higher meant that the difference between the standard and comparison duration signals had to be at least as big as the JND for a participant to report high confidence. In other words, those with a criterion estimate > 1 based their confidence judgment almost exclusively on the perceptual discriminability between the two test durations, as assumed by our model. Those with a criterion estimate < 1, though, reported high confidence even when the perceptual difference between the two test durations was smaller than the JND, indicating that their confidence judgment was also influenced by other factors that we did not include in our model.

## Discussion

In this study, we used confidence judgments to probe the perceptual nature of the effect of the speed profile of a visual object on the apparent duration of a subsecond interval that contains it. The two experiments we described here yielded a similar pattern of results. First, we found that the confidence estimates of perceived duration were affected by stimulus speed in a similar way as discrimination estimates: An interval containing a stationary stimulus appeared substantially shorter than that containing a stimulus drifting at a constant rate, whereas intervals that embedded linearly accelerating or decelerating stimuli appeared more mildly compressed. However, duration estimates extracted from confidence judgments were overall higher than those extracted from discrimination judgments. Second, the precision of both discrimination and confidence judgments did not change across speed conditions. Third, an orthogonal regression analysis revealed that the discrimination and confidence measures of perceived duration were positively correlated, but the hypothesis that the regression lines coincided with the equality line was not strongly supported across conditions. Finally, we described an observer model that assumes that both the discrimination and confidence judgments depend on duration discriminability, which is extracted from the discrimination judgment, but the latter also require an additional step, where this information is compared against an internal criterion. The model predicted all of the variance in the real precision estimates for the discrimination judgments. For the confidence judgments, over 75% of the variance in the real data was predicted by the model, which takes the internal confidence criterion as the only free parameter. A clear pattern of inter-individual differences in the confidence criterion estimates in our dataset was highlighted by the predictions of our model, revealing a distinction between those participants that based their judgments entirely on duration discriminability and those who did not.

In both Experiments 1 and 2, we replicated the previous finding that the duration of an interval containing a stationary visual object appears substantially compressed when compared to the duration of an interval containing a temporally modulated sensory stimulus (Brown, 1931; Goldstone & Lhamon, 1974; Kanai et al., 2006; Kaneko & Murakami, 2009; Lhamon & Goldstone, 1974; Roelofs & Zeeman, 1951). An interval with an accelerating visual pattern was previously reported to appear compressed relative to an interval containing a stimulus that changes over time at a constant rate (Binetti et al., 2012; Binetti et al., 2020; Bruno et al., 2015; Matthews, 2011; Sasaki et al., 2013), as we observed here, in both experiments. As far as the perceived duration of an interval containing a decelerating pattern is concerned, the reported effects differ across studies: Some of them have reported duration compression (Matthews, 2011; Matthews, 2013), and other studies have reported no change or mild dilation (Binetti et al., 2012; Bruno et al., 2015). Here, we used similar stimuli and procedure as in a previous study (Bruno et al., 2015), where the differences in perceived duration between intervals with accelerating and decelerating visual objects were substantially more pronounced than those reported here, as acceleration induced strong duration compression (up to ∼30% for a 600-ms interval), whereas deceleration induced only a very mild expansion (less than ∼10%). The lack of replication of the magnitude (and direction, in the case of deceleration) of these effects in the present study may be ascribed to the different speed range used here for the accelerating and decelerating conditions. In fact, in the condition eliciting the largest difference in apparent duration in Bruno et al. (2015), the minimum speed was 0 and the maximum speed was 20°/s (average speed, 10°/s), whereas, in the present study we used a lower maximum speed of 10°/s (average speed, 5°/s) to make sure that the stimuli were displayed online without distortions. In the same study, the authors showed that the magnitude of the duration changes was contingent on the speed range rather than acceleration or deceleration per se, as it did not change when they kept the initial and final speeds constant and varied the standard stimulus duration.

More generally, the results of Experiment 2, where we used a wider comparison duration range and where participants received feedback about their performance in the practice trials, replicated the pattern observed in Experiment 1 for both perceived duration and precision. Previous reports showed that people are aware of mistakes in their decisions even without explicit feedback (Rabbitt, 1966; Yeung & Summerfield, 2012). In addition to that, we showed here that participants did not seem to use feedback to calibrate their confidence. In fact, in both experiments, the curve heights at the PMCs were substantially smaller than 1 (Figures 2 and 4), indicating a tendency to overestimate the correctness of very difficult judgments, which was previously reported for perceptual decisions (Baranski & Petrusic, 1994; Baranski & Petrusic, 1999; Bruno & Baker, 2021; Harvey, 1997). The same studies also reported a tendency to underestimate the correctness of very easy judgments, which we also observed for very short and, especially, very long durations. Previous studies suggest that personality traits (Pallier, Wilkinson, Danthiir, Kleitman, Knezevic, Stankov, & Roberts, 2002), but not cognitive styles (Blais, Thompson, & Baranski, 2005), play a role in over- and underconfidence biases in discrimination tasks. Interestingly, in the present study, the simulated observers generated by our model showed an even larger overconfidence (curve height at PMC = 0.624 ± 0.022) than the real participants (0.68 ± 0.03), with a main effect of participant type, *F*(1, 52) = 8.172, *p* = 0.006 (data not shown), which might suggest that sensory noise contributed to the overconfidence effect more than the participants’ personality.

The mild but significant bias in perceived duration observed in the drifting condition, where no change was expected because standard and comparison had the same speed profile, was mainly due to a tendency to report very long comparison durations as being longer than the standard duration more often than reporting very short durations (with the same absolute distance from the center of the range) as being shorter. In fact, although the proportion of “comparison longer” responses for the longest comparison duration was smaller than 1, participants’ performance was at chance when the two tests had the same duration (see Figures 2 and 4). This bias, too, did not disappear either with the feedback provided in Experiment 2 or by extending the duration range to include extremely long and extremely short durations. The fact that the confidence estimates were also shifted the same amount argues against a decisional bias. As the direction of the said effect was opposite to that observed for the main effects of the present study (i.e., duration overestimation vs. underestimation), it is unlikely that it created any form of confound. Also, we showed that the pattern of results remained the same when we used the drifting condition as baseline, indicating that the differences between the discrimination and confidence measures were not exaggerated by comparing them against the standard duration. We can only surmise that having randomized both the presentation order of the two tests and their relative spatial location might have played a role, as it seems clear that participants perceived the very long duration as being longer than the standard duration, but somehow they attributed it to the wrong location. If, for example, we assume a memory buffer that stores the duration of the first test interval, together with the location of the embedded stimulus, to compare it with the duration of the second test (and its location), then this buffer might have a limited capacity, and when that is nearly exceeded (e.g., when one of the test intervals is very long) then duration information is favored relative to spatial information, which might be more easily forgotten.

The speed profile of our stimuli had a similar impact on how both discrimination and confidence judgments estimated perceived duration. For both, we observed stronger duration compression for the stationary stimuli than for the accelerating and decelerating stimuli. The mean PMCs for confidence were slightly misaligned relative to the mean PSEs for discrimination, though, indicating that discrimination judgments were affected by both perceptual and decisional processes (Gallagher et al., 2019). Orthogonal regression helped us further analyze the relationship between our two measures of central tendency at the individual level by testing the equivalence of the two measures. It showed that a positive orthogonal correlation existed between our two measures of perceived duration for all of the speed conditions, but for most of our speed conditions the slope and intercept of the regression lines differed from 1 and 0, respectively. In their paper on the effect of pursuit eye movements on perceived background motion, Luna et al. (2021) observed a similar pattern, where a linear regression analysis revealed that their measures of central tendency for discrimination and confidence were positively correlated, but the best fit was never the equality line. As their mean estimates of central tendency for discrimination and confidence did not differ, they concluded that this positive correlation further argued against the existence of a decision bias. In the present study, the two mean estimates are instead different (Figures 3a and 5a); therefore, we cannot exclude the influence of decisional processes, but the positive (orthogonal) correlations between the individual PSEs and PMCs indicate a more predominant perceptual component.

The observation that the effect of speed profile on discrimination and confidence measures of perceived duration followed a similar pattern suggests that both types of judgments were informed by sensory information. However, to decide whether one has high or low confidence in the correctness of their discrimination judgment, the sensory difference in the two durations has to be compared against an internal criterion. We modeled the duration information associated with each test interval with sensory noise. We assumed that a simple comparison between the two resulting duration signals was enough to formulate a discrimination judgment. For the confidence judgment, the same comparison had to be weighted by the JND (i.e., an approximation of *d*′) and compared against a confidence criterion, modeled as a threshold to exceed to formulate a high confidence judgment. Note that the assumption of a separate criterion for confidence did not entail the two judgments being based on different types of sensory information, as suggested by some studies (De Martino, Fleming, Garrett, & Dolan, 2013; Fleming, Ryu, Golfinos, & Blackmon, 2014; Li, Hill, & He, 2014). In fact, our model assumed that both types of judgment are based on duration discriminability and are therefore affected by the same sensory noise.

The idea that we need a separate criterion to determine how confident we are in our decisions has been previously proposed in different forms. In a recent study, Arnold et al. (2021), measured changes in perceived orientation and precision after adaptation to contrast-modulated Gabors using both discrimination and confidence judgments. Their results were well predicted by a labeled-line observer model (consisting of several channels, each maximally responding to a given stimulus orientation) that assumed that the two judgments were based on different magnitudes (i.e., different criterions) of the same kind of sensory information. This means that a high confidence judgment would require a larger sensory difference between the stimuli than that required to formulate a discrimination judgment. Mamassian and de Gardelle (2021) proposed a generative model based on signal detection theory that contains both a sensory criterion and a confidence criterion and assumes that a confidence decision is affected by both sensory and confidence noise.

The main difference between these models and ours is that we included the confidence criterion as the only free parameter to predict confidence judgments, equaling the assumption that confidence decision are based on duration discriminability and sensory noise. The discrimination judgment, on the other hand, was modeled to be entirely based on duration discriminability. Overall, between 75% and 80% of the variance in our real data was captured by this simple assumption (Figure 7). More importantly, the predictions of our model allowed us to highlight a clear pattern of inter-individual differences in weighting sensory evidence to form a confidence judgment. In fact, the model explained virtually all of the variance (all *R*^2^ > 0.915) in the real data for participants with a predicted criterion higher than 1 (Figure 8). This value is not arbitrary, as a criterion of 1 or higher indicates that, to have high confidence, the difference between the two duration signals (which are affected only by sensory noise, according to our model) has to be at least as large as the JND between the two durations. Therefore, participants with a criterion higher than 1 based their confidence judgments on the same sensory information they used for their discrimination judgments, and, in fact, their FWHHs were very well captured by our model. For these participants, the confidence criteria ranged between 1 and 4. This finding indicates that, as shown by Arnold et al. (2021) for tilt perception, high confidence in a perceptual decision requires a different magnitude of the same sensory information (i.e., a larger difference in duration between the two test intervals relative to the JND). Also, it shows that individual participants set their internal thresholds at different distances (in sensory units) from the JND, pointing to a tendency to be more conservative or less conservative in their confidence criterion. It is worth stressing that, even though we did not include a random component to account for this tendency, our model was still able to capture this variability. In fact, it predicted participants’ FWHHs equally well when the estimated confidence criterion was substantially larger than 1.

On the other hand, participants with a criterion lower than 1 tended to report high confidence even when the difference between the two duration signals was smaller than the JND, implying that their judgment was also affected by other components that we did not include in our model. The correlations between the individual JNDs and confidence criteria (data not shown) reached statistical significance for participants with a criterion higher than 1 but not for those with a criterion smaller than 1, further suggesting that confidence judgments in these two groups are affected by different factors or computations. Our model could account for about 70% of the variance in the data of participants with a criterion estimate lower than 1. One possibility is that the rest of the variance might be explained by sensory factors that are not used for the discrimination decision. In fact, Mamassian and de Gardelle (2021) proposed that there might be some additional sensory information used to form a confidence judgment that would be acquired after the perceptual decision and would boost the participants’ confidence (they refer to this component as “confidence boost,” to distinguish it from “confidence noise,” which would instead reduce confidence). Alternatively, the remaining variance could be due to non-sensory noise components that have been shown to specifically affect confidence judgments (Shekhar & Rahnev, 2021). Bang, Shekhar, and Rahnev (2019) recently showed that, perhaps counterintuitively, higher levels of sensory noise in a perceptual task can lead to higher metacognitive efficiency, measured using meta-*d*′, which is the ratio between the signal and a combination of sensory and confidence noise, and *M_ratio_*, which is the ratio of meta-*d*′ and *d*′ (Maniscalco & Lau, 2012). They suggested that this finding supports the idea that confidence judgments are affected by independent metacognitive noise. If that is the origin of our unexplained variance, it would be interesting to investigate why only some participants are affected by this confidence noise but other participants (i.e., those with a confidence criterion > 1) do not seem to show this influence.

In two studies where biases were induced in perceptual appearance with either adaptation or by manipulating the prior statistics of the presented stimuli, Caziot and Mamassian (2021) showed that confidence judgments, which aligned well with the perceptual reports (indicating that both judgments were based on the same sensory evidence), were modulated more by the subjective sensory distance of the test stimulus (i.e., the distance in sensory units of the stimulus from the participant's PSE) than by its objective sensory distance (i.e., the distance in sensory units of the stimulus from the physical equality of the two tests). In our model, we took the distance of the simulated duration signal for the comparison interval from that of the standard (see Modeling section), which, in the framework of Caziot and Mamassian, would correspond to the objective sensory distance, as the subjective sensory distance would correspond to the distance of the SDS for the comparison from the PSE. One of the predictions that might be drawn from Caziot and Mamassian's observation is that the performance of our model in predicting the real confidence data should be worse for the conditions where the average difference between the PSE and the physical equality between comparison and standard durations was larger (e.g., in the stationary condition, where the mean perceived duration was substantially shorter than 500 ms). This did not seem to be the case. In fact, the amount of variance explained by our model in the stationary condition did not differ from that captured in the other conditions (see Figure 7b). However, it must be noted that here we only focused on predicting the FWHH for the confidence curves, whereas Caziot and Mamassian focused their analysis on their measures of central tendency.

More generally, we did not intend for our model to account for all aspects of confidence. More complex models, such as those cited above, can do that much better. We were mainly interested in seeing how much variability in our dataset we could explain with the fewest assumptions. We believe our very simple model served this purpose quite well. We showed that, for about half of our participants, the only assumption of a confidence criterion based solely on sensory information was enough to account for all of the individual variability. It must be noted that our model makes assumptions only on our estimates of precision but not on those of central tendency. Therefore, it can only make sensible predictions regarding the link between the discrimination JNDs and the confidence FWHHs. Future developments of the model will include both a content-based (Johnston, 2010; Johnston, 2014; Roseboom et al., 2019) explanation of the speed-related changes in perceived duration and a decisional noise component to account for the differences between discrimination and confidence estimates of central tendency.

## Conclusions

We showed here that the effect of stimulus speed on apparent duration contains both perceptual and decisional components. The perceptual component was substantially more pronounced. We proposed a simple observer model that assumes that the same type of sensory information informs both discrimination and confidence judgments and that the latter require an internal criterion based on discriminability. The criterion estimates revealed a clear pattern of inter-individual differences between those participants who relied entirely on perceptual differences to rate their confidence and those who also used information that did not influence their discrimination judgments.
